# Smartphone use in a large US adult population: Temporal associations between objective measures of usage and mental well-being

**DOI:** 10.1073/pnas.2427311122

**Published:** 2025-10-13

**Authors:** Ari Winbush, Daniel McDuff, John Hernandez, Andrew Barakat, Allen Jiang, Conor Heneghan, Benjamin W. Nelson, Nicholas B. Allen

**Affiliations:** ^a^Department of Psychology, University of Oregon, Eugene, OR 97403; ^b^Google Research, Seattle, WA 98109; ^c^Google Research, Mountain View, CA 94043; ^d^Verily Life Sciences, Dallas, TX 75019

**Keywords:** mental health, smartphones, wellbeing, mobile sensing

## Abstract

Smartphones are essential tools in daily life yet concerns persist about their potential effects on mental health and well-being. Research on these relationships often relies on subjective measures of smartphone use, which can lack validity. These studies are also often limited by small, homogenous samples and the inability to differentiate between types of smartphone activities (e.g., social media vs. other applications). In this study, we analyzed 250,000 d of smartphone usage data from 10,099 adults, using objective measures of smartphone usage collected over 4 wk. We examined normative usage patterns and the temporal relationships between smartphone use and mood. Our findings reveal weak or null associations between smartphone use and mood, both cross-sectionally and longitudinally, and at both individual and group levels. Demographic factors, such as age and gender, were stronger predictors of mood than smartphone use. Statistically significant results that were observed showed that younger adults demonstrated a stronger association between social media use and lower mood cross-sectionally, but not longitudinally. Non-social app use was linked to lower mood in between-subjects analyses, but better mood in within-subjects analyses. However, all effects were small (or null), indicating minimal practical significance. These findings suggest that smartphone use has a limited or negligible impact on mood and well-being over a 4-wk period and do not support claims of a strong or causal relationship between smartphone use and mental well-being across this timeframe.

## Significance

Smartphones are an indispensable tool in everyday life; however, there are concerns about how their use may impact mental health and well-being. Our analysis of a quarter of a million days of objective smartphone usage across over 10,000 diverse adult participants reveals little evidence for strong bidirectional associations between mental well-being and smartphone use.

Smartphones are pervasive tools that have become integrated into everyday tasks including shopping, communication, navigation, entertainment, social networking, and tracking finances and health. Approximately 83.7% or 6.7 billion people own smartphone devices worldwide (1) with 85% of Americans owning a smartphone device (2). As a result, these devices have become ubiquitous and indispensable (3). The possible impact of digital device use on mental health and well-being is a pressing question to which individuals, families, schools, policy makers, legislators, and digital designers are all demanding answers (4). Several recent authoritative reviews have made urgent calls for future research projects to address this gap in knowledge (5–7). One critical aim of research in this field is therefore to identify which patterns of smartphone use are associated with benefits versus risks, and who is more vulnerable to harmful versus beneficial outcomes. This can inform evidence-based product design, education, and regulation aimed at maximizing benefits and minimizing risks of smartphones and other digital devices.

Yet, critical questions remain about the normative patterns of smartphone use in everyday life. How often do adults use their phones? How much smartphone “screen time” is typical or atypical? How much screen time is consumed by apps that are used for different services/categories? What is the relationship between these behavior patterns and well-being, and in particular, what are the temporal associations between smartphone use and these outcomes? Do the answers to these questions differ by individual differences such as gender and age?

A key limiting factor in providing reliable answers to these questions is that much of the literature to date around smartphone usage has relied on self-reported measures (8), which have questionable validity and precision. Research has generally demonstrated null to weak associations between self-reported and objective measures of smartphone use (9–11). For example, a study of 187 adolescents found participants’ self-report of the total time spent on smartphones exceeded the objective data by around 760 min per week (12). In several other studies, overall or specific types of smartphone usage were also overestimated (10, 12–14). These studies are of relatively small cohorts [N = 187 (12), N = 50 (14)] or collect data only from specific apps [e.g., WhatsApp data only; (13)] making it difficult to draw conclusions about how the results generalize to the broader population or overall smartphone use. A systematic review found low correlations between self-reported and objectively logged digital media usage (15) and other studies have replicated these findings for total smartphone use, social media use (10, 12), and phone pickups and behavior (11). Further, studies employing objective measures have reported inconsistent relationships between smartphone usage and well-being (16–18). These discrepancies mean studies relying on self-reported measures can lead to erroneous conclusions regarding the impact of actual smartphone usage on mental health symptoms (18).

There are other methodological issues that are likely to contribute to the lack of precision in estimating these effects. These include, examining within-person vs between-person effects (19), examining temporal relationships between smartphone use and well-being metrics [especially whether smartphone use prospectively predicts changes in mental health symptoms, or whether mental health symptoms predict changes in smartphone use; (20)] and separately estimating use of different types of applications on the smartphone (e.g., social media versus non–social media applications). The latter issue is especially salient given the speculation that social media use is particularly harmful, and that harm may be more likely in particular demographic groups (4, 21). The importance of studying social media specifically has also been emphasized by the United States Surgeon General’s advisory on social media in 2023 (22) highlighting the perceived risks that this presents to young people in particular, and proposing greater transparency about the impact. More recently, an opinion piece in the New York Times called for a warning label for social media (23). One of the recommendations of the Surgeon General’s report was that “technology companies could better and more transparently assess the impact of their products, share data with independent researchers to increase our collective understanding of the impacts.” To this end, in this study university researchers collaborated with researchers at a technology company (Google) to conduct a large-scale study that would have been logistically challenging (if not impossible) to perform without the help of an industry partner. As noted, there remains ambiguity about the temporal relationship between smartphone use and mental well-being. For example, does increased smartphone use precede changes in mental well-being or does lower well-being lead to changes in smartphone use, or neither? Despite assertions of a causal relationship between smartphone use and poorer mental well-being, a previous longitudinal study using self-report of smartphone usage found that while social-media use did not predict depressive symptoms over time for males or females, greater depressive symptoms predicted more frequent social-media use only among adolescent girls (20). Answering this question is challenging as it requires making valid causal inferences leveraging longitudinal real-world data that accurately measure smartphone use and mental well-being across time.

In this paper, we present data on normative patterns of smartphone usage from a large intensive longitudinal study in adult participants. Each participant contributed up to 4 wk of longitudinal data during which objective measures of smartphone use and mood were recorded on a daily basis. We analyze a quarter of a million days of smartphone usage data from 10,099 subjects from a diverse sample of US adults across all 50 states. We aimed to answer the following questions: What is the typical duration of screen time, how often do people unlock their smartphones, and how do objective patterns of smartphone usage vary with self-reported measures of well-being concurrently and prospectively? We specifically investigated associations between social versus non-social applications and examined the direction of prediction by evaluating whether smartphone use showed a prospective (i.e., week to week) association with subsequent changes in mental well-being, or whether mental well-being has a prospective association with changes in smartphone use (4, 21). Finally, we also separately examined between- and within-person effects, and whether demographic factors such as age and gender moderated the association between smartphone use and well-being.

## Results

### Descriptive Data on Objective Patterns of Smartphone Use.

**Fig. 1.**
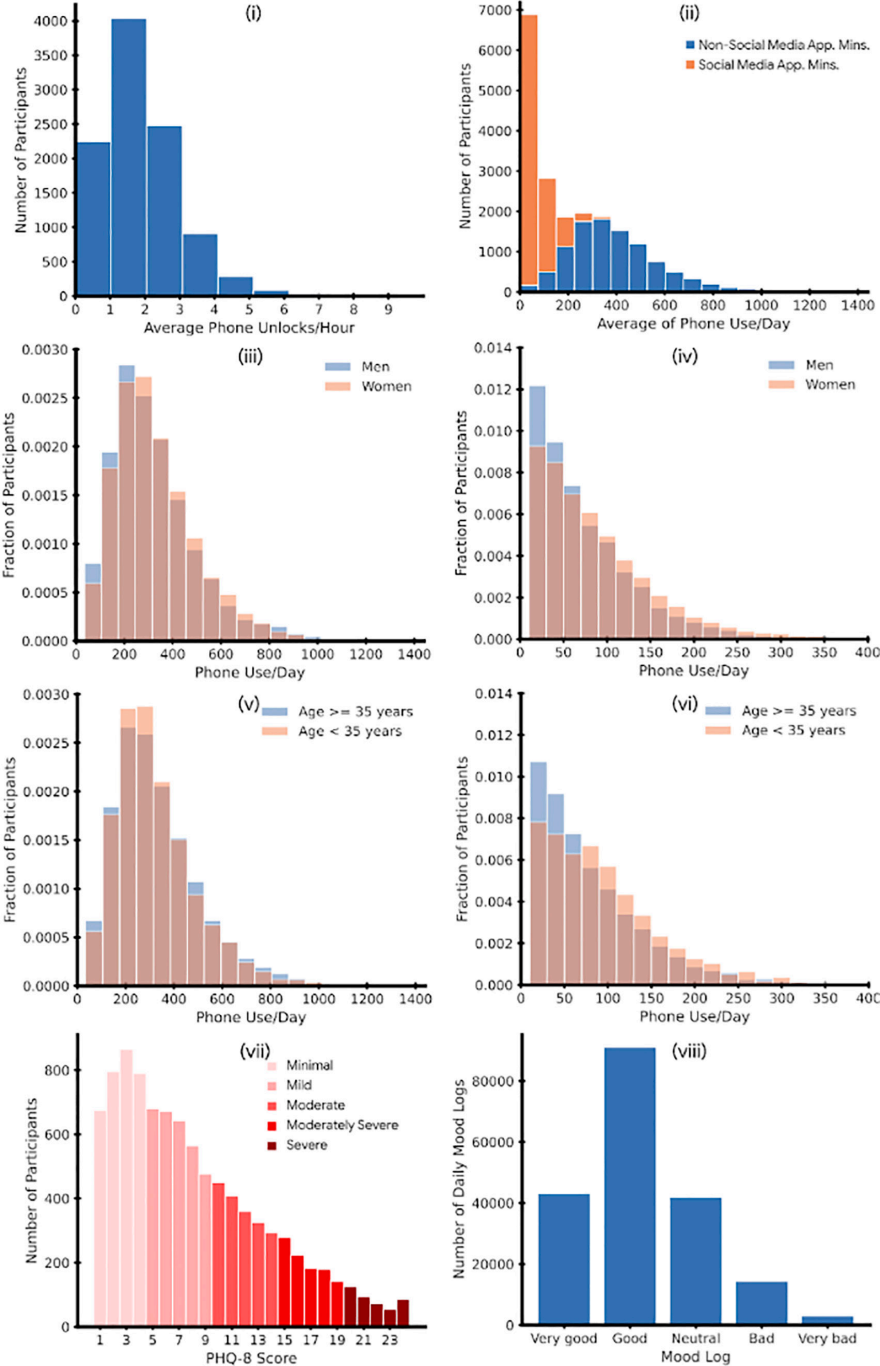
(*i*) Average number of phone unlocks per hour, (*ii*) Average number of minutes of application use per hour, (*iii*) overlapping histograms showing non–social media application use by gender, (*iv*) overlapping histograms showing social media application by gender, (*v*) overlapping histograms showing non–social media application use by age, (*vi*) overlapping histograms showing social media application by age, (*vii*) Distribution of PHQ-8 scores, (*viii*) Distribution of daily mood logs for participants in our study.

The broad distributions of key parameters highlight the highly variable range of smartphone use patterns across the study population. The majority of users had their smartphone on and unlocked the device repeatedly throughout the day (Fig. 1). Median unlocks per hour were 1.71 (per day = 41; daily range = 0 to 344) and median minutes of application time across all applications per hour were 15.2 (per day = 364; daily range = 0 to 1,440).

The median minutes of screen time for all non–social media applications per day was 295 (daily range = 0 to 1,440). The median minutes of screen time on all social media applications per day was 42.7 (daily range = 0 to 624). However, the distribution was heavily skewed with a long tail and many people spending upward of 300 min per day on social media applications.

Table 1 summarizes smartphone use statistics, number of mood ratings completed, distributions of daily mood ratings, and baseline depressive symptoms (PHQ-8). The distribution of mood logs was skewed with the median daily mood log of “Good.” PHQ-8 scores for the population followed an expected positive skew in a nonclinical, community based sample, with a median score of 6 and 32.5% of the population scored 10 or higher indicating evidence of moderate or severe depressive symptoms (24) (Table 1 and Fig. 1).

**Table 1.** Descriptive Statistics

Factor (Level)	Mean (SD)	Median (25th to 75th)
Phone Use		
Average Duration/Hour (Mins)	16.3 (7.68)	15.16 (10.8 to 20.6)
Average Social Media Duration/Hour (Mins)	2.60 (2.81)	1.78 (0.32 to 3.98)
Average Phone Unlocks/Hour	1.89 (1.13)	1.71 (1.10 to 2.49)
Daily Check-Ins		
Number Mood Ratings Completed	19.3 (7.90)	22 (15 to 26)
Average Mood Rating (1to 5)	3.77 (0.65)	3.36 (3.81 to 4.17)
PHQ-8		
Score	5.82 (4.11)	5 (2 to 8)

Phone usage, daily check-in, and PHQ-8 mean, SD, median (50th), 25th, and 75th percentiles.

### Smartphone Use Predicting Mood.

#### Social-app usage predicting mood.

##### Concurrent week.

No effects were observed when examining associations between social-app usage and mood across all study participants (between subjects) or in the context of social-app usage relative to one’s own usual pattern (within subjects).

##### Subsequent week.

Likewise, no significant associations were observed when examining associations between social-app usage and mood during the following week in between-subject or within-subject analyses. However, there was a small but statistically significant moderating effect between age and social-app usage in the between-subjects analysis, indicating that increased social-app usage in a given week was more strongly associated with lower mood during the following week in younger participants.

#### Non-social app usage predicting mood.

##### Concurrent week.

Higher non-social-app usage was associated with lower mood in the same week across the study population (between-subjects β: 0.060; 95% CI [0.029 to 0.091], *P* <0.001 (Table 2). However, the reverse relationship was found in the within-subjects analysis where higher non-social-app usage relative to one’s own baseline was associated with more positive mood in the same week (β: −0.025; 95% CI [−0.035 to −0.015], *P* <0.001). A moderating effect of age was found with younger subjects exhibiting a greater effect of non-social-app usage on mood (between-subjects β: −0.001; 95% CI [−0.002 to 0.001]; *P* =0.025). Females also had a stronger association between non-social-app usage and lower mood during the same week relative to males (β: 0.043; 95% CI [0.001 to 0.076]; *P* =0.014).

**Table 2.** Model results for analyses predicting mood by smartphone usage

Predicting:	Subsequent-Weekly Mood from:						Concurrent-Weekly Mood from:					
	Social App Usage			Non-Social App Usage			Social App Usage			Non-Social App Usage		
Model Predictor	β	SE	*P*-Val	β	SE	*P*-Val	β	SE	*P*-Val	β	SE	*P*-Val
*Age*	−0.012	0.001	**<0.001**	−0.013	0.001	**<0.001**	−0.014	0.001	**<0.001**	−0.014	0.001	**<0.001**
*Gender [Female vs Male(ref)]*	0.109	0.022	**<0.001**	0.111	0.021	**<0.001**	0.115	0.020	**<0.001**	0.114	0.020	**<0.001**
*Gender [Genderqueer/Transgender vs Male(ref)]*	0.330	0.052	**<0.001**	0.320	0.051	**<0.001**	0.315	0.051	**<0.001**	0.309	0.050	**<0.001**
*Between-Subjects social-app usage*	0.018	0.019	0.363				0.008	0.018	0.646			
*Within-Subjects social-app usage*	0.003	0.007	0.679				0.006	0.006	0.393			
*Between-Subjects social-app usage × Age*	0.002	0.001	**0.043**				0.001	0.001	0.077			
*Between-Subjects social-app usage × Gender [Female vs Male(ref)]*	−0.013	0.022	0.550				0.004	0.019	0.844			
*Between-Subjects social-app usage × Gender [Genderqueer/Transgender vs Male(ref)]*	−0.002	0.043	0.968				0.031	0.040	0.435			
*Between-Subjects social-app usage × Within-subjects social-app usage*	−0.001	0.003	0.677				0.001	0.003	0.665			
*Between-Subjects non-social-app usage*				0.051	0.017	**0.002**				0.060	0.016	**<0.001**
*Within-Subjects non-social-app usage*				−0.025	0.007	**<0.001**				−0.025	0.005	**<0.001**
*Between-Subjects non-social-app usage × Age*				−0.001	0.001	0.055				−0.001	0.001	**0.025**
*Between-Subjects non-social app usage × Gender [Female vs Male(ref)]*				0.040	0.018	**0.028**				0.043	0.017	**0.014**
*Between-Subjects non-social-app usage × Gender [Genderqueer/Transgender vs Male (ref)]*				−0.020	0.046	0.670				−0.035	0.042	0.403
*Between-Subjects non-social app usage ×*												
*Within-subjects non-social app usage*				0.008	0.004	0.062				0.004	0.004	0.259
**R^2^**	0.033			0.039			0.038			0.046		

##### Subsequent week.

Model results examining whether non-social-app usage is predictive of mood during the subsequent week were similar to the concurrent week models (Table 2). Higher non-social-app usage was associated with lower mood during the subsequent week across the study population (between-subjects β: 0.051; 95% CI [0.018 to 0.084], *P* = 0.002). In contrast, higher non-social-app usage relative to one’s own baseline was associated with better mood (within-subjects β: −0.025; 95% CI [−0.039 to, −0.011], *P* <0.001). Similar to concurrent-week models, females had a stronger association between non-social-app usage and lower mood (between-subjects β: 0.040; 95% CI [0.005 to 0.075], *P* =0.028).

### Mood Predicting Smartphone Use.

#### Mood predicting social-app-usage.

##### Concurrent week.

No significant associations were observed between mood and social-app usage during the same week (between-subjects or within-subjects; Table 3).

**Table 3.** Model results for analyses predicting smartphone usage by mood

	Subsequent Week						Concurrent Week					
Using Weekly Mood to Predict:	Social App Usage			Non-Social App Usage			Social App Usage			Non-Social App Usage		
Model Predictor	β	SE	*P*-Val	β	SE	*P*-Val	β	SE	*P*-Val	β	SE	*P*-Val
*Age*	−0.014	0.001	**<0.001**	0.002	0.001	**0.044**	−0.014	0.001	**<0.001**	0.001	0.001	0.122
*Gender [Female vs Male(ref)]*	0.318	0.022	**<0.001**	0.069	0.022	**0.002**	0.321	0.022	**<0.001**	0.073	0.022	**0.001**
*Gender [Genderqueer/Transgender vs Male(ref)]*	0.316	0.057	**<0.001**	0.221	0.058	**<0.001**	0.306	0.056	**<0.001**	0.233	0.058	**<0.001**
*Between-Subjects daily check-in score*	−0.001	0.010	0.938	0.020	0.012	0.087	0.013	0.010	0.175	0.018	0.010	0.085
*Within-Subjects daily check-in score*	−0.005	0.005	0.298	−0.012	0.005	**0.033**	0.007	0.004	0.111	−0.009	0.005	0.066
*Between-Subjects daily check-in score × Age*	0.001	4e-04	0.070	3e-04	4e-04	0.428	−3e-04	3e-04	0.363	−1e-04	4e-04	0.733
*Between-Subjects daily check-in score × Gender [Female vs Male(ref)]*	−5e-04	0.010	0.965	0.010	0.011	0.352	0.003	0.008	0.731	0.009	0.010	0.385
*Between-Subjects daily check-in score × Gender [Genderqueer/Transgender vs Male(ref]]*	0.033	0.028	0.235	0.009	0.027	0.749	0.027	0.025	0.283	−0.012	0.025	0.629
*Between-Subjects daily checkin-score × Within-Subjects daily check-in score*	0.001	0.003	0.745	0.001	0.003	0.831	−0.001	0.002	0.680	0.001	0.003	0.794
**R^2^**	0.053			0.003			0.054			0.003		

##### Prospective associations with subsequent week.

No significant predictive associations were seen between mood and social-app usage during the subsequent week (between-subjects or within-subject; Table 3).

#### Mood predicting non-social-app-usage.

##### Concurrent week.

No significant associations were seen between mood and non-social-app usage during the same week in either the between-subjects and within-subjects categories (Table 3).

##### Prospective associations with the subsequent week.

Patterns were similar to the same-week analysis. When each participant’s weekly mood was compared to their own baseline, better individual weekly mood predicted higher non-social-app usage the following week (within-subjects β: −0.012; 95% CI [−0.022 to −0.002]; *P* =0.033).

##### Exploring the effect of time-frame.

In order to see whether the time frame of the prospective analyses would significantly impact the associations between app usage and daily mood rating, we also conducted a set of models based on day-to-day prediction (instead of week-to-week as in the models presented here). These are presented in the (*SI Appendix*, Table S4). Overall, the day-to-day analyses showed a strong congruence with the findings of the week-to-week analyses, suggesting that the patterns of association described here are consistent across these two different time frames. One notable exception was the finding of a significant association between a participant’s daily mood relative to the whole population (between-subjects effect) and non-social app usage the following day with more negative mood predicting greater non-social app usage the following day. Given this strong consistency, we have chosen to present week-to-week analyses as our main findings.

### Associations Between Demographic Factors, Phone Use, and Mood.

In all of our models we found consistent associations between demographic (age and gender) factors and smartphone usage patterns and mood. For models where mood was the primary dependent variable, there was a negative association between age and average daily check-in scores; i.e., younger age was associated with lower mood. For example, age was a significant predictor in our model examining the effects of social-app usage and mood during the subsequent week (β: −0.012; 95% CI [−0.014 to −0.010], *P* <0.001; Table 2). Likewise with regard to gender, there was a significant association for both females and individuals identifying as genderqueer with lower mood as compared to males. These associations were present in both the same and subsequent-week models (Table 2).

For models where smartphone usage was the primary dependent variable, age was negatively associated with social-app usage, and positively associated with non-social-app usage. Note for example the effects of age in our subsequent week-models for social-app usage (β: −0.014; 95% CI [−0.016 to -0.012]; *P* <0.001; Table 3) and non-social-app usage (β: 0.002; 95% CI [4e-05 to 0.004]; *P* =0.044; Table 3). This trend was consistent across both model paradigms, indicating that younger subjects tended toward higher levels of social-app usage. In terms of gender, being female or identifying as genderqueer was associated with higher usage of both the social and non-social-app categories. These trends were consistent across both the concurrent and subsequent-week models (Table 3).

## Discussion

We conducted one of the the largest studies to date (n = 10,099) investigating the relationships between objectively measured smartphone use and self-reported measures of mood in adults over a 4-wk period. Demographic associations with mood and device use were generally consistent with previous findings, and suggest that younger adults, females, and genderqueer individuals experience lower self-reported mood and used social media more (25–28). However, we observed generally null or weak associations (i.e., small effect sizes) between objectively measured patterns of device use and daily ratings of mood, and those associations that were observed reflected a mix of associations with both positive and negative mood outcomes. Among the findings that did reach statistical significance, we observed that although there were no main effects showing an association between use of social media applications and mood either cross-sectionally or prospectively, younger participants showed a stronger association between social media use and mood the subsequent week than did older participants. Between-subjects models indicated that, the use of non-social applications was cross-sectionally associated with lower mood but was associated with better mood when participant’s use of non-social applications was compared to their own personal baseline (i.e., within subjects models). This pattern was replicated for prospective analyses, with non-social app usage showing a prospective association with poorer mood in between participant analyses, and an association with more positive mood in within-participant analyses. So, in general, non-social app usage was only associated with poorer mood when usage levels exceeded the average compared to other participants, while higher usage relative to one’s own baseline was associated with more positive mood.

A critical issue in interpreting these findings is not just statistical significance, but also the range of effect sizes observed, which indicate the importance of the findings for drawing actionable conclusions. While our study did find some statistically significant associations between app usage and mood, it is crucial to consider not only the low frequency with which significant effects were observed, but also their magnitude (29). In large studies, small and inconsequential effects often reach statistical significance. For instance, our model showed a significant association between non-social app usage and mood the following week. To illustrate the effect size of this association, we fitted this model to one of our participants with an average hourly non-social app use of 19 min, corresponding to a weekly mood score of 0 (neutral mood). All of our imputed models predicted that this individual would need to increase their hourly usage by 70 min per hour (which is, of course, technically impossible) to shift their mood score to 1 (i.e., bad mood). This equates to an extra 18+ h of daily app use, assuming usage is spread across their waking hours. Clearly such a dramatic increase is unrealistic (none of our participants even approached this level of usage). Therefore, the effects we observed are so small that they require implausibly large behavioral changes to produce even minor mood shifts of one point on our rating scale. This is further reflected in the low explanatory power of our models, with the variance explained indicating that factors like age and gender play a far more substantial role in predicting mood.

Another important factor is the variability in the direction of effects across the between and within-subjects analyses, and across social and non-social app usage. Social-app usage was not associated with lower mood either cross-sectionally or prospectively). Although some previous studies have shown small cross-sectional associations between social media use and mood (30, 31), they were primarily based on self-report measurement, which, as we outlined in the introduction, has methodological limitations. Furthermore, the lack of a prospective association between social app usage and subsequent mood also supports critiques indicating that it is not justified to draw strong causal inferences about these associations based on cross-sectional data alone (31, 32). Moreover, the associations between non-social app usage and mood were highly variable, with between-subjects effects showing associations with lower mood, and within-subjects effects showing associations with more positive mood. This potentially contradictory pattern of findings has been observed in other types of data and is sometimes referred to as Simpson’s paradox (33). For example, in the case of the association between exercise and cardiac arrest, at the population level, more exercise is associated with lower rates of cardiac arrest, but at the individual level, people are more likely to have cardiac arrest while exercising. Similarly, these findings suggest that while people who use non-social apps more than others may be more likely to have a lower mood (both concurrently and prospectively), using apps more than you usually do is associated with a better mood. Because our study revealed predominantly small or null effect sizes, and because the associations between non-social app usage and mood differed between between-subjects and within-subjects analyses, replication of these findings is crucial before drawing strong conclusions. The data suggest that individuals who generally use non-social apps more frequently might also tend to experience lower mood. However, this does not imply that reducing individual usage of non-social apps will improve mood; in fact, it could potentially worsen it. To definitively determine causality, experimental designs will be needed. It is worth noting that a recent meta-analysis of experimental manipulations of social media use, specifically within-person manipulations, found no evidence for effects on mental health (34). Therefore, these findings are more consistent with the conclusion that app usage and mood are likely not causally related.

In terms of understanding normative patterns of smartphone use as revealed by objective measurement, smartphone usage in our study as measured by daily screen time (mean 391 min) was somewhat higher than several recent studies (11, 16–18, 27, 35–38) but lower than other studies (11). Daily social media use in our study (mean 62 min) is comparable to several recent studies (27, 37, 39) but much lower than reported by Felisoni and colleagues (40). In general, our results are consistent with the finding that smartphone usage has increased since the COVID-2019 pandemic (27) and may also reflect demographic factors such as our predominantly female population (68%) with a high proportion of college graduates (59%).

Although this study has many strengths, including our large sample size, rich longitudinal data over a 4-wk period (including objective measures of smartphone use), and modeling methods that addressed temporal relationships as well as both between and within-subjects effects, there are some important limitations that should be borne in mind when interpreting these findings. First, although our sample is large and diverse, there is still underrepresentation of some groups, including males and some traditionally underrepresented racial and ethnic groups—although it should be noted that the absolute size of these subgroups is still larger than most studies. Furthermore, the study was limited to individuals who use an Android smartphone as their personal device, which could have introduced some demographic and other biases (41), although the large and diverse sample that was recruited does mitigate against this concern somewhat (Table 4). A particularly important limitation is that the participants consisted of only adults (18+), limiting the generalizability of these findings to younger adolescence—a sensitive period of brain development, identity formation, and heightened sensitivity to social information, which may include smartphone use (33, 42). As such, the current study cannot draw conclusions about these questions in younger teenagers, which has been a significant focus of the public debate (43, 44). In general, examining these issues within truly representative samples remains a challenge for the field. Future studies targeting adolescent populations and underrepresented demographic groups are necessary to understand the unique interplay between individual differences, device use, and mental well-being. Second, although we have been able to examine temporal dynamics across days and weeks, we cannot rule out the possibility of cumulative or delayed effects that might emerge over longer time frames (i.e., months or years). Future research should employ longitudinal designs to investigate whether prolonged smartphone use may affect mental well-being. Third, this study relied on the categorization of apps provided by the Google Play Store, which are generally determined by the developers. While generally accurate, this system may not always reflect an app’s primary function or user experience. For example, Snapchat, often considered a social media app, is listed under communication apps in the Play store, and therefore was not included in the social app category. This is perhaps understandable given that most time on Snapchat is spent communicating with friends as opposed to observing content (e.g., photos, videos), which may justify the characterization of Snapchat as a messaging app (45). The applications that were included in the social category were primarily “broadcasting” or “one to many” apps, where one’s posts are generally observable to all users of the platform, not a specific recipient (as is typically the case with Snapchat). Finally, while temporal modeling of associations between app usage and mood does allow from stronger causal inferences to be drawn than does purely cross-sectional studies, it should be noted that these analyses are still correlations, and strong experimental designs are required to provide the strongest evidence for causal inference (46).

**Table 4.** Demographic data of study population

Factor (Level)	N	%
**Age**		
18 to 29	1,263	12.5%
30 to 49	5,828	57.8%
50 to 69	2,677	26.4%
70+	338	3.3%
Not Specified	3	0.03%
**Gender**		
Female	6,860	67.9%
Male	2,869	28.4%
Queer/Nonconforming	282	2.5%
Trans	77	0.8%
Not Specified	11	0.1%
**Orientation**		
Heterosexual	7,818	77.4%
LGBTQIA+	1,885	18.7%
I Prefer Something Else	384	3.8%
Not Specified	12	0.1%
**Race**		
Caucasian	8,435	83.5%
Black	462	4.6%
Asian	297	2.9%
Native American/Alaskan Native	104	1.0%
Native Hawaiian/Pacific Islander	18	0.1%
Multiracial/Other	537	5.3%
Not Specified	246	2.4%
**Ethnicity**		
Not Hispanic or Latino	9,272	91.8%
Hispanic or Latino	804	8.0%
Not Specified	23	0.2%
**Income**		
Are Comfortable	4,396	43.5%
Have Just Enough to Get Along	4,197	41.6%
Can’t Make Ends Meet	1,474	14.6%
Not Specified	32	0.3%
**Highest Level of Education**		
High School	2,389	23.7%
Associates	1,601	15.9%
Bachelors	3,055	30.3%
Graduate School	2,898	28.7%
Not Specified	156	1.5%
**Disability**		
Yes	1,791	17.7%
No	7,912	78.3%
Prefer not to answer	353	3.5%
Not Specified	43	0.4%
Total	10,099	100%

Counts and distributions (%) of participants by demographic factors for the 10,099 participants in our study.

Despite the sometimes vociferous discourse surrounding smartphone and social media use, mental health, and well-being, our results show little evidence of short-term impact of smartphone use on mood (a key indicator of mental well-being) from week to week or of mood on smartphone use over the same timescale. Moreover, among the findings that were statistically significant, we observed a mix of associations with both negative and positive mood outcomes. Our findings are largely consistent with other published reports of negligible (i.e., very small effect sizes) and/or nonsignificant relationships between objective smartphone usage patterns and well-being measures (11, 18, 21, 47). They are also consistent with recent meta-analyses showing small or null relationships between smartphone use and mood among adults (30, 46) and adolescents (48, 49), and with studies suggesting that other determinants of mood are likely to have much stronger effects (50). Indeed, it is probably best to think of smartphones as a context in which a wide variety of experiences occur, and as such, further research should focus on the factors that increase or reduce the likelihood of beneficial versus harmful online experiences, rather than the use of smartphones or particular categories of applications per se.

## Materials and Methods

### Data.

The data for this study were collected as part of a prospective, observational study to investigate patterns and relationships between digital device use patterns, including sensor data from phones reflecting both behavioral and physiological processes, and self-reported measures of mental health and well-being. The study protocol (51) involved collection of passive sensing from an individual’s personal Android smartphone, and optionally a wearable (Fitbit), for the complete 4-wk study period. In this work, we focus analysis on the phone data. Participants were recruited via posts that were made on social media platforms, leveraging the reach and audience of the University of Oregon and Google accounts. Also, in app-notifications were made in the Fitbit app inviting users to participate in the study. As noted in the protocol paper, recruitment was metered throughout the study to ensure we were able to recruit a diverse and representative sample. (N.B. Only Android users were enrolled as our software for collecting objective metrics of phone use was specific to the Android platform.) The highest level of education of the participants was High School (23.7%), Associates Degree (15.9%), Bachelor’s Degree (30.2%), Graduate Degree (28.7%). The remaining 1% did not report any of these. The reported financial situation of the participants was comfortable (43.5%), having just enough to get along (41.6%) and not being able to make ends meet (14.6%). While not perfectly representative of the broader US population, our dataset reflects a diverse range of socioeconomic participants.

All study data were collected with informed consent, and participants were asked to complete an assurance of understanding quiz to ensure understanding. The data were deidentified and all 18 HIPAA identifiers (name, email, address, etc.) were removed. Passive data collection began only after participants completed all onboarding surveys. Data were collected in a manner that inherently restricts the granularity and detail of participant information to protect privacy. For example, data on application usage were aggregated by hour and by category of app, rather than individual apps. Location information was collected semantically rather than precisely such that investigators would not know participants’ geographical locations, only whether they spent time at certain semantic locations of interest.

#### Participants.

**Fig. 2.**
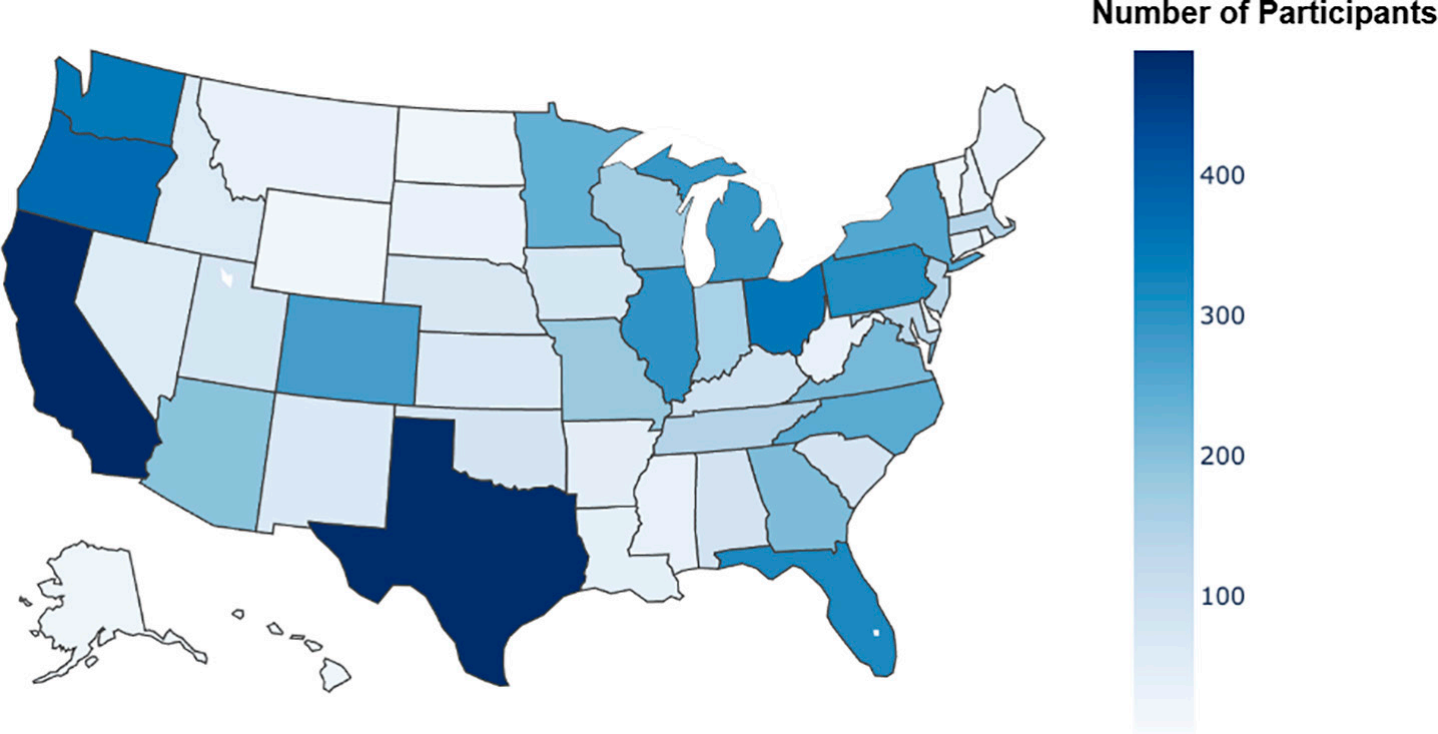
The distribution of participants by home state (in number of participants).

Following the research protocol, we recruited 10,099 participants into the 4-wk study. Study demographics and the geographic distribution of participants across the United States are shown in Table 4 and Fig. 2, respectively. We were able to sample participants with age diversity (mean = 44 y, min=18, max = 92) and had participation from all 50 US states.

#### Mobile data.

Patterns of device use were measured objectively via the study mobile app using Android APIs (52). The four categories of phone activity measurement include:

**Phone Unlocks.** The number of times the phone was unlocked was counted per hour of each day.
**Screen Time.** The amount of screen time is measured as the amount of time the phone was unlocked and an app was in the foreground. It is measured in microseconds and aggregated per hour of each day as per the user consent. From screen time we derive three measures:
**Total Screen Time.** Total screen time is defined as the minutes per hour in which the phone is unlocked with an app in the foreground.
**Total Session Length.** Total session length is defined as the number of minutes per hour for which an app category is used when it is used.
**Average session length.** Average session length is defined as the number of minutes per hour, which an app category is used when it is used, divided by the number screen unlocks in the hour.

#### Total Application Category Time.

Application usage is quantified for categories of apps rather than individual apps, and application categories were determined by those listed on the Google Play Store and chosen by developers of the study app.

#### Application categories.

Mobile application usage time was logged by category rather than specific application name to preserve privacy. Applications were categorized according to their primary description on the Android Play Store according to the following categories: business/productivity, communication, education, entertainment, health, lifestyle, music, news, social, travel, and video categories. In this paper, we specifically analyze non-social application usage and social application usage (see *SI Appendix* for a list of the specific applications that were included in the “social” category).

#### Surveys.

##### Pre- and postsurveys.

Demographics questionnaire and the Patient Health Questionnaire (PHQ-8) (24) were collected at baseline [online with other measures; (51)]. The PHQ-8 has good internal consistency [Cronbach’s alpha = 0.87; (53)].

##### Daily check-in questionnaires.

Throughout the 4 wk of the study, participants received a single question pushed to their phone each day at 8 am. The single question asked “In general, how have you been feeling over the last day?” and the participant will respond using a single-select 5-item Likert scale that ranges between “very bad” and “very good”. Responses are scored for downstream modeling on a 5-point range centered at 0 (neutral) and ranging from -2 (very good) to 2 (very bad). Therefore, a higher check-in score corresponds to a lower mood.

#### Ethics reviews and compensation.

##### Human subjects research ethics review.

The Institutional Review Board (IRB) of the University of Oregon approved the study protocol. Our study design also went through several rounds of ethical reviews from the Responsible AI, Health Ethics, Privacy, Security and Legal teams at Google. Further, an external advisory panel was assembled with Mental Health experts from outside of Google, to solicit their input and feedback on the study design.

##### Compensation.

The conditions to be eligible for study compensation required the participants to: 1) consent and enable sensor collection at study start, 2) complete the prestudy assessments, 3) complete a minimum cumulative of 7 d (1 wk) of daily status assessments and to 4) complete the poststudy assessments. Participants who completed all required elements of the study were eligible to enter a raffle for a $50 gift card.

##### Modeling.

Our modeling primarily focused on phone usage metrics (social and non-social app usage, phone unlocks, and session length), and daily check-in scores. Data were aggregated weekly, calculating the mean values for each metric over a 1-wk period (168 h). Missing data from sensing modalities required at least 48 complete hours of data within a study week for averages to be calculated; otherwise, the data were treated as missing and set to not-a-number (NaN). Weekly mood ratings were derived by calculating the rounded mean of daily check-in scores.

To examine variability, both Within-Subject and Between-Subject variables were calculated for each metric. Within-Subject variables were centered on a participant’s baseline average across all time points, while Between-Subject variables were centered on the overall baseline average across participants (54, 55) with the demographic variable, age, also mean-centered.

We used multilevel regression models R v.4.41 using the “lme4”package [v. 1.1-35.5; (56)] with maximum likelihood estimation to test associations between smartphone usage (social and non-social app categories, session length, and phone unlocks) and mood. Models examined both concurrent (same-week) and temporal (subsequent-week) relationships across a 4-wk data collection period. This design resulted in three repeated measures for subsequent-week models and four repeated measures for same-week models.

The primary analysis focused on associations between app usage and mood, with supplementary models exploring session length and phone unlocks (*SI Appendix*, *Supplemental* Tables S1*a* and S2*b* and *Supplemental Results* A). Additional daily-level models examined associations between daily app usage/mood and mood/app usage the following day, providing a comparison to the weekly models (*SI Appendix*, *Supplemental* Tables S3 and S4 and *Supplemental Results* B).

For weekly models, all variables represent the standardized weekly mean of hourly values, while daily models use standardized daily means and check-in scores.

#### Missing data imputation.

The survey instruments were scored and missing values were imputed using the mean of the scores on the items that were responded to before summing the values. For the PHQ-8 the subject needed to have answered at least half of the questions otherwise the corresponding survey score was treated as missing. (PHQ_8 scores were only used to describe sample characteristics and were not used in statistical modeling. Only 9 participants (0.08%) had more than three missing items on the PHQ-8.

For daily check-in questionnaire responses, each day of the study exhibited a proportion of participants that failed to complete the daily check-in (*SI Appendix*, Fig. S1). Calculation of weekly check-in score means was restricted to participants who provided check-in responses for at least 4 d during the week of interest. If a participant failed to meet this criterion, their corresponding mean check-in score was treated as missing for that particular week. Missing weekly check-in score means were estimated using multiple imputation with demographic variables, intake PHQ-8 scores, phone social-app usage, phone unlocks, and nonmissing mean check-in scores as predictors. Demographic variables including auxiliary variables were race, gender, sexual orientation, age-group, disability status, education level, marital status, and financial situation. For models using weekly mean check-in values as predictors of phone app usage, weekly means of Within-Subject and Between-Subject check-in scores were imputed using predictive mean matching in R using the “mice” package (57, 58). For models using phone usage variables as predictors, weekly means of check-in scores were imputed using ordered logit methods. A total of 40 imputations were generated for each model and fitted model parameter estimates were pooled using Rubin’s rules. All results reported in tables represent pooled standardized parameter estimates. For supplemental models examining day to day effects of phone usage and daily check-in scores, raw check-in scores were imputed using demographic and auxiliary variables and daily average phone social-app usage and unlocks as predictors and utilizing ordered logit methods. In order to maintain consistent coverage of all phone use variables across all participants, day to day modeling was restricted to the first 23 d of the study.

## Supplementary Material

Appendix 01 (PDF)

## Data Availability

Some study data available (The dataset used in these analyses, containing phone usage metrics, mood logs and demographics for participants in the Google Digital Wellbeing Study will be available to researchers. The data are deidentified and will only be shared with approved researchers who complete a dataset access request form and agree to the data usage policies. Upon receipt of this form, a link to the dataset will be provided to the named researcher. Data access form: https://docs.google.com/forms/d/1_DyQN8DFr7DeSm6RZ6q0Eei4DUes5KyEmnYR07pYLdY/edit) (59).

## References

[1] S. O’Dea, Number of smartphone users worldwide from 2016 to 2029 (2024). https://www.statista.com/statistics/330695/number-of-smartphone-users-worldwide/.(accessed 2 September 2025).

[2] J. Poushter, C. Huang, L. Silver, Internet, Smartphone and Social Media Use in Advanced Economies 2022 (Pew Research Center, 2022).

[3] G. K. Gupta, Ubiquitous mobile phones are becoming indispensable. ACM Inroads. **2**, 32–33 (2011).

[4] J. Haidt, N. Allen, Scrutinizing the effects of digital technology on mental health. Nature **578**, 226–227 (2020). 32042091 10.1038/d41586-020-00296-x

[5] C. L. Odgers, M. R. Jensen, Annual research review: Adolescent mental health in the digital age: Facts, fears, and future directions. J. Child Psychol. Psychiatry **61**, 336–348 (2020). 31951670 10.1111/jcpp.13190 PMC8221420

[6] A. Wongkoblap, M. A. Vadillo, V. Curcin, Researching mental health disorders in the era of social media: Systematic review. J. Med. Internet Res. **19**, e228 (2017). 28663166 10.2196/jmir.7215 PMC5509952

[7] S. Chancellor, M. De Choudhury, Methods in predictive techniques for mental health status on social media: A critical review. NPJ Digit. Med. **3**, 43 (2020). 32219184 10.1038/s41746-020-0233-7 PMC7093465

[8] J. C. Levenson, A. Shensa, J. E. Sidani, J. B. Colditz, B. A. Primack, Social media use before bed and sleep disturbance among young adults in the United States: A nationally representative study. Sleep. **40**, zsx113 (2017). 28934521 10.1093/sleep/zsx113 PMC8205627

[9] J. Boase, R. Ling, Measuring mobile phone use: Self-report versus log data. J. Comput. Mediat. Commun. **18**, 508–519 (2013).

[10] T. Mahalingham, P. M. McEvoy, P. J. F. Clarke, Assessing the validity of self-report social media use: Evidence of no relationship with objective smartphone use. Comput. Human Behav. **140**, 107567 (2023).

[11] P. Coyne, J. Voth, S. J. Woodruff, A comparison of self-report and objective measurements of smartphone and social media usage. Telemat. Inform. Rep. **10**, 100061 (2023).

[12] P. H. Lee, A. C. Y. Tse, C. S. T. Wu, Y. W. Mak, U. Lee, Validation of self-reported smartphone usage against objectively-measured smartphone usage in Hong Kong Chinese adolescents and young adults. Psychiatry Investig. **18**, 95–100 (2021). 10.30773/pi.2020.0197 PMC7960745 33517618

[13] C. Montag , Smartphone usage in the 21st century: Who is active on WhatsApp?. BMC Res. Notes. **8**, 331 (2015). 26238512 10.1186/s13104-015-1280-z PMC4522968

[14] T. Deng , Measuring smartphone usage and task switching with log tracking and self-reports. Mob. Media Commun. **7**, 3–23 (2019).

[15] D. A. Parry , A systematic review and meta-analysis of discrepancies between logged and self-reported digital media use. Nat. Hum. Behav. **5**, 1535–1547 (2021). 34002052 10.1038/s41562-021-01117-5

[16] D. A. Ellis, Are smartphones really that bad? Improving the psychological measurement of technology-related behaviors. Comput. Human Behav. **97**, 60–66 (2019).

[17] D. Rozgonjuk, J. C. Levine, B. J. Hall, J. D. Elhai, The association between problematic smartphone use, depression and anxiety symptom severity, and objectively measured smartphone use over one week. Comput. Hum. Behav. **87**, 10–17 (2018).

[18] H. Shaw , Quantifying smartphone ‘use’: Choice of measurement impacts relationships between ‘usage’ and health. Technol. Mind Behav. **1**, 1–14 (2020).

[19] C. A. Stamatis , Differential temporal utility of passively sensed smartphone features for depression and anxiety symptom prediction: A longitudinal cohort study. NPJ Ment. Health Res. **3**, 1 (2024). 38609548 10.1038/s44184-023-00041-y PMC10955925

[20] T. Heffer, M. Good, O. Daly, E. MacDonell, T. Willoughby, The longitudinal association between social-media use and depressive symptoms among adolescents and young adults: An empirical reply Clin. Psychol. Sci. **7**, 462–470 (2019).

[21] A. Orben, A. K. Przybylski, S. J. Blakemore, R. A. Kievit, Windows of developmental sensitivity to social media. Nat. Commun. **13**, 1649 (2022). 35347142 10.1038/s41467-022-29296-3 PMC8960761

[22] Office of the Assistant Secretary for Health (OASH), Surgeon General Issues New Advisory About Effects Social Media Use Has on Youth Mental Health (U.S. Department of Health and Human Services, 2023).

[23] V. H. Murthy, Surgeon general: Why I’m calling for a warning label on social media platforms (2024.). https://www.nytimes.com/2024/06/17/opinion/social-media-health-warning.html. (accessed 2 September 2025).

[24] K. Kroenke , The PHQ-8 as a measure of current depression in the general population. J. Affect. Disord. **114**, 163–173 (2009). 18752852 10.1016/j.jad.2008.06.026

[25] J. D. Alexander , Passively sensing smartphone use in teens with rates of use by sex and across operating systems. Sci. Rep. **14**, 17982 (2024). 39097657 10.1038/s41598-024-68467-8 PMC11297944

[26] S. L. Reisner, S. L. Katz-Wise, A. R. Gordon, H. L. Corliss, S. B. Austin, Social epidemiology of depression and anxiety by gender identity. J. Adolesc. Health. **59**, 203–208 (2016). 27267142 10.1016/j.jadohealth.2016.04.006 PMC4958506

[27] K. Chemnad , Smartphone usage before and during COVID-19: A comparative study based on objective recording of usage data. Informatics. **9**, 98 (2022).

[28] S. Horwood, J. Anglim, S. R. Mallawaarachchi, Problematic smartphone use in a large nationally representative sample: Age, reporting biases, and technology concerns. Comput. Human Behav. **122**, 106848 (2021).

[29] P. Ranganathan, C. S. Pramesh, M. Buyse, Common pitfalls in statistical analysis: Clinical versus statistical significance. Perspect. Clin. Res. **6**, 169–170 (2015). 26229754 10.4103/2229-3485.159943 PMC4504060

[30] J. Hancock, S. X. Liu, M. Luo, H. Mieczkowski, Psychological well-being and social media use: A meta-analysis of associations between social media use and depression, anxiety, loneliness, eudaimonic, hedonic and social well-being. SSRN Electron. J. **45**, 101294 (2022).

[31] Z. Vahedi, A. Saiphoo, The association between smartphone use, stress, and anxiety: A meta-analytic review. Stress Health. **34**, 347–358 (2018). 29673047 10.1002/smi.2805

[32] A. H. M. Bradley, A. L. Howard, Stress and mood associations with smartphone use in university students: A 12-week longitudinal study. Clin. Psychol. Sci. **11**, 921–941 (2023). 37694230 10.1177/21677026221116889 PMC10491487

[33] C. H. Wagner, Simpson’s paradox in real life. Am. Stat. **36**, 46 (1982).

[34] C. J. Ferguson, Do social media experiments prove a link with mental health: A methodological and meta-analytic review. Psychol. Pop. Media. **14**, 201–206 (2024).

[35] S. M. Jones-Jang , Good news! Communication findings may be underestimated: Comparing effect sizes with self-reported and logged smartphone use data. J. Comput.-Mediat. Commun. **25**, 346–363 (2020).

[36] J. Ohme, T. Araujo, C. H. de Vreese, J. T. Piotrowski, Mobile data donations: Assessing self-report accuracy and sample biases with the iOS screen time function. Mob. Media Commun. **9**, 205015792095910 (2021).

[37] S. Prasad , A study of magnitude and psychological correlates of smartphone use in medical students: A pilot study with a novel telemetric approach. Indian J. Psychol. Med. **40**, 468–475 (2018). 30275623 10.4103/IJPSYM.IJPSYM_133_18 PMC6149309

[38] S. Alshakhsi , Problematic internet usage: The impact of objectively recorded and categorized usage time, emotional intelligence components and subjective happiness about usage. Heliyon **8**, e11055 (2022). 36281419 10.1016/j.heliyon.2022.e11055 PMC9587279

[39] D. D. Felisoni, A. S. Godoi, Cell phone usage and academic performance: An experiment. Comput. Educ. **117**, 175–187 (2018).

[40] H. Shaw, D. A. Ellis, L. R. Kendrick, F. Ziegler, R. Wiseman, Predicting smartphone operating system from personality and individual differences. Cyberpsychol. Behav. Soc. Netw. **19**, 727–732 (2016). 27849366 10.1089/cyber.2016.0324

[41] R. E. Dahl, N. B. Allen, L. Wilbrecht, A. B. Suleiman, Importance of investing in adolescence from a developmental science perspective. Nature. **554**, 441–450 (2018). 29469094 10.1038/nature25770

[42] G. Hartwell, M. Gill, M. Zenone, M. McKee, Smartphones, social media, and teenage mental health. BMJ **385**, e079828 (2024). 38806185 10.1136/bmj-2024-079828

[43] V. A. Goodyear , Approaches to children’s smartphone and social media use must go beyond bans. BMJ **388**, e082569 (2025). 40147838 10.1136/bmj-2024-082569

[44] J. M. Vaterlaus, K. Barnett, C. Roche, J. A. Young, ‘Snapchat is more personal’: An exploratory study on Snapchat behaviors and young adult interpersonal relationships. Comput. Human Behav. **62**, 594–601 (2016).

[45] A. Bleske-Rechek, K. M. Morrison, L. D. Heidtke, Causal inference from descriptions of experimental and non-experimental research: Public understanding of correlation-versus-causation. J. Gen. Psychol. **142**, 48–70 (2015). 25539186 10.1080/00221309.2014.977216

[46] A. Orben, Teenagers, screens and social media: A narrative review of reviews and key studies. Soc. Psychiatry Psychiatr. Epidemiol. **55**, 407–414 (2020). 31925481 10.1007/s00127-019-01825-4

[47] P. M. Valkenburg, A. Meier, I. Beyens, Social media use and its impact on adolescent mental health: An umbrella review of the evidence. Curr. Opin. Psychol. **44**, 58–68 (2022). 34563980 10.1016/j.copsyc.2021.08.017

[48] E. J. Ivie, A. Pettitt, L. J. Moses, N. B. Allen, A meta-analysis of the association between adolescent social media use and depressive symptoms. J. Affect. Disord. **275**, 165–174 (2020). 32734903 10.1016/j.jad.2020.06.014

[49] M. Panayiotou, L. Black, P. Carmichael-Murphy, P. Qualter, N. Humphrey, Time spent on social media among the least influential factors in adolescent mental health: Preliminary results from a panel network analysis. Nat. Ment. Health. **1**, 316–326 (2023).

[50] D. McDuff , The Google Health Digital Well-Being Study: Protocol for a Digital Device Use and Well-Being Study. JMIR Res Protoc. **13**, e49189 (2024). 38743938 10.2196/49189 PMC11134241

[51] Google, Android API reference. https://developer.android.com/reference (accessed 2 September 2025).

[52] J. Arias Torre , Reliability and cross-country equivalence of the 8-item version of the patient health questionnaire (PHQ-8) for the assessment of depression: Results from 27 countries in Europe. Lancet Reg. Health. **31**, 100659 (2023). 10.1016/j.lanepe.2023.100659 PMC10272490 37332385

[53] C. K. Enders, D. Tofighi, Centering predictor variables in cross-sectional multilevel models: A new look at an old issue. Psychol. Methods. **12**, 121–138 (2007). 17563168 10.1037/1082-989X.12.2.121

[54] P. J. Curran, D. J. Bauer, The disaggregation of within-person and between-person effects in longitudinal models of change. Annu. Rev. Psychol. **62**, 583–619 (2011). 19575624 10.1146/annurev.psych.093008.100356 PMC3059070

[55] D. Bates, M. Mächler, B. Bolker, S. Walker, Fitting linear mixed-effects models using lme4. J. Stat. Softw. **67**, 1–48 (2015).

[56] S. van Buuren, K. Groothuis-Oudshoorn, Mice: Multivariate imputation by chained equations in R. J. Stat. Softw. **45**, 1–67 (2011).

[57] K. J. Lee, J. A. Simpson, Introduction to multiple imputation for dealing with missing data. Respirology. **19**, 162–167 (2014). 24372814 10.1111/resp.12226

[58] J. Kropko, B. Goodrich, A. Gelman, J. Hill, Multiple imputation for continuous and categorical data: Comparing joint multivariate normal and conditional approaches. Polit. Anal. **22**, 497–519 (2014).

[59] A. Winbush , Data from “Smartphone use in a large US population: Temporal associations between objective measures of usage and mental well-being.” Google Forms. https://docs.google.com/forms/d/1_DyQN8DFr7DeSm6RZ6q0Eei4DUes5KyEmnYR07pYLdY/edit. Deposited 22 September 2025. 10.1073/pnas.2427311122 PMC12582163 41082655

