# Supplementary material for: Smartphone use in a large US adult population: Temporal associations between objective measures of usage and mental well-being
Source: Proc Natl Acad Sci U S A. 2025 Oct 13;122(43):e2427311122. doi: 10.1073/pnas.2427311122 (PMC12582163; doi:10.1073/pnas.2427311122)
Supplement: Supplementary file 1 — Appendix 01 (PDF) [file pnas.2427311122.sapp.pdf]

Supplementary Analyses: Winbush et al., Smartphone Use in a Large US Adult Population: Temporal Associations Between Objective Measures of Usage and Mental Wellbeing

Supplemental Results A: Average Session Length and Phone Unlocks

The Relationship between Session Lengths and Mood

Predicting Mood with Social-App Session Length (Table 1a)

Concurrent Week
No associations between social-app session length and mood during the concurrent week were observed across study participants (Between Subjects). Likewise, no effects were observed with respect to social-app session length relative to one's own baseline (Within-Subjects).

Prospective Associations with Subsequent week

Longer social-app session length (Between Subjects) was associated with lower mood (β: 0.052; 95% CI [0.011 to 0.093], $p=0.012$) with no other associations noted.

Predicting Mood with non-Social-App Session Length (Table 1a)

Concurrent Week
Results were largely the same as the main results examining non-social-app usage. Longer non-social-app session lengths were associated with lower mood during the same week across the study population (Between Subjects: β: 0.084; 95% CI [0.051 to 0.117], $p<0.001$). When examining non-social-app session length relative to one's own baseline (Within-Subjects), the reverse relationship was observed (β: -0.023; 95% CI [-0.033 to -0.013], $p<0.001$). A moderating effect of age was also found with younger subjects exhibiting a greater effect of non-social-app session length on mood (β: -0.002; 95% CI [-0.004 to -4e-05], $p=0.002$).

Prospective Associations with Subsequent week

Longer non-social-app session lengths were associated with lower mood across the population (Between Subjects: β: 0.083; 95% CI [0.048 to 0.118], $p<0.001$) with the reverse relationship observed when examining this variable relative to one's own baseline (Within-Subjects; β: -0.026; 95% CI [-0.040 to -0.012], $p<0.001$).

Supplemental Table 1a. Model Results For Analyses Predicting Mood by Smartphone Use Session Length

Predicting:	Subsequent-Weekly Mood from:						Concurrent-Weekly Mood from:					
Average Session Length:	Social App Usage			Non-Social App Usage			Social App Usage			Non-Social App Usage		
**Model Predictor**	**β**	**SE**	**p-Val**	**β**	**SE**	**p-Val**	**β**	**SE**	**p-Val**	**β**	**SE**	**p-Val**
Age	-0.012	0.001	**<0.001**	-0.014	0.001	**<0.001**	-0.014	0.001	**<0.001**	-0.015	0.001	**<0.001**
Gender [Female vs Male(ref)]	0.097	0.022	**<0.001**	0.101	0.021	**<0.001**	0.105	0.020	**<0.001**	0.105	0.020	**<0.001**
Gender [Genderqueer/Transgender vs Male(ref)]	0.317	0.052	**<0.001**	0.303	0.051	**<0.001**	0.304	0.051	**<0.001**	0.291	0.050	**<0.001**
Between-Subjects social-app usage	0.052	0.021	**0.012**				0.035	0.019	0.068			
Within-Subjects social-app usage	-0.007	0.008	0.365				-0.001	0.007	0.888			
Between-Subjects social-app usage × Age	0.001	0.001	0.413				2e-04	0.001	0.758			
Between-Subjects social-app usage × Gender [Female vs Male(ref)]	-0.026	0.023	0.241				4e-04	0.020	0.985			
Between-Subjects social-app usage × Gender [Genderqueer/Transgender vs Male(ref)]	-0.028	0.045	0.532				0.014	0.041	0.736			
Between-Subjects social-app usage × Within-Subjects social-app usage	-0.001	0.003	0.575				-1e-04	0.002	0.966			
Between-Subjects non-social app usage				0.083	0.018	**<0.001**				0.084	0.017	**<0.001**
Within-Subjects non-social app usage				-0.026	0.007	**<0.001**				-0.023	0.005	**<0.001**
Between-Subjects non-social app usage × Age				-0.002	0.001	**0.004**				-0.002	0.001	**0.002**
Between-Subjects non-social app usage × Gender [Female vs Male(ref)]				0.021	0.020	0.284				0.030	0.019	0.103
Between-Subjects non-social app usage × Gender [Genderqueer/Transgender vs Male (ref)]				-0.033	0.047	0.474				-0.037	0.043	0.379

Between-Subjects non-social app usage ×<br>*Within-Subjects non-social app usage*		0.004	0.004	0.345			-3e-04	0.003	0.926
**R^2**	0.034	0.041			0.039		0.048		

The Relationship between Mood and Session Lengths (Supplemental Table 1b)

Association with Social-App Session Length

Concurrent Week

No significant associations between Within-Subject or Between-Subject mood and social-app session length were observed during the concurrent week period.

Prospective Associations with Subsequent week

Similar to concurrent week models, no significant associations between Within-Subject or Between-Subject mood and social-app session length during the subsequent week were observed.

Association with Non-Social-App Session Length

Concurrent Week

Better mood was associated with shorter non-social-app session length across the study population (Between Subjects: β: 0.024; 95% CI [0.006 to 0.042]; p=0.007).

Prospective Associations with Subsequent week

Results were similar to models examining concurrent week associations. Across the study population, better mood was predictive of shorter non-social-app session length (Between-Subjects; β: 0.024; 95% CI [0.002 to 0.046]; p=0.026). The opposite was observed for Within-Subjects weekly mood (β: -0.013; 95% CI [-0.023 to -0.003]; p=0.012).

Supplemental Table 1b. Model Results For Analyses Predicting Smartphone Use Session Length by Mood

	Subsequent Week(Average Session Length)						Concurrent Week (Average Session Length)					
**Using Weekly Mood to Predict:**	**Social App Usage**			**Non-Social App Usage**			**Social App Usage**			**Non-Social App Usage**		
**Model Predictor**	**β**	**SE**	**p-Val**	**β**	**SE**	**p-Val**	**β**	**SE**	**p-Val**	**β**	**SE**	**p-Val**
Age	-0.005	0.001	**<0.001**	0.011	0.001	**<0.001**	-0.005	0.001	**<0.001**	0.011	0.001	**<0.001**
Gender [Female vs Male(ref)]	0.339	0.022	**<0.001**	0.148	0.022	**<0.001**	0.345	0.022	**<0.001**	0.152	0.022	**<0.001**
Gender [Genderqueer/Transgender vs Male(ref)]	0.366	0.057	**<0.001**	0.329	0.058	**<0.001**	0.373	0.057	**<0.001**	0.345	0.057	**<0.001**
Between-Subjects daily check-in	0.010	0.012	0.416	0.024	0.011	**0.026**	0.018	0.010	0.078	0.024	0.009	**0.007**
Within-Subjects daily check-in	-0.008	0.006	0.162	-0.013	0.005	**0.012**	0.004	0.005	0.380	-0.008	0.004	0.055
Between-Subjects daily check-in × Age	0.001	4e-04	0.109	2e-04	4e-04	0.518	-1e-04	3e-04	0.693	-1e-04	3e-04	0.833
Between-Subjects daily check-in × Gender [Female vs Male(ref)]	0.002	0.011	0.880	0.004	0.010	0.702	0.008	0.009	0.376	0.003	0.009	0.723
Between-Subjects daily check-in × Gender [Genderqueer/Transgender vs Male(ref]]	0.051	0.029	0.081	0.017	0.024	0.486	0.025	0.027	0.352	-0.012	0.022	0.605
Between-Subjects daily check-in × Within-Subjects daily check-in	-0.002	0.003	0.561	-1e-04	0.003	0.986	-5e-04	0.003	0.850	-0.001	0.002	0.701
**R^2**	0.031			0.022			0.032			0.022		

The Relationship between Phone-Unlocks and Mood (Supplemental Table 2a)

Concurrent Week
Higher phone-unlocks was associated with better mood during the same week across study participants (Between Subjects: β: -0.069; 95% CI [-0.096 to -0.042]; *p*<0.001) while higher phone-unlocks relative to one's own baseline (Within-Subjects) was not significantly associated lower mood .

Prospective Associations with Subsequent week
Phone unlocks were predictive of mood during the subsequent week with results similar to models examining concurrent week associations. Higher Between-Subject phone unlocks were associated with better mood the following week (β: -0.069; 95% CI [-0.100 to -0.038]; *p*<0.001). The opposite effect was observed when examining the association between Within-Subject phone unlocks and subsequent-week mood (β: 0.018; 95% CI [0.004 to 0.032]; *p*=0.006).

Supplemental Table 2a. Model Results For Analyses Predicting Mood by Smartphone Unlock Counts

Predicting:	Subsequent-Weekly Mood from Phone-Unlocks			Concurrent-Weekly Mood from Phone-Unlocks		
**Model Predictor**	**β**	**SE**	**p-Val**	**β**	**SE**	**p-Val**
Age	-0.015	0.001	**<0.001**	-0.016	0.001	**<0.001**
Gender [Female vs Male(ref)]	0.095	0.021	**<0.001**	0.101	0.020	**<0.001**
Gender [Genderqueer/Transgender vs Male(ref)]	0.304	0.051	**<0.001**	0.298	0.051	**<0.001**
Between-Subjects phone-unlocks	-0.069	0.016	**<0.001**	-0.069	0.014	**<0.001**
Within-Subjects phone-unlocks	0.018	0.007	**0.006**	0.002	0.005	0.755
Between-Subjects phone-unlocks × Age	0.001	0.001	0.164	0.001	0.001	0.373
Between-Subjects phone-unlocks × Gender [Female vs Male(ref)]	-0.006	0.018	0.751	-0.008	0.017	0.625
Between-Subjects phone-unlocks × Gender [Genderqueer/Transgender vs Male(ref)]	0.009	0.048	0.858	-0.033	0.045	0.462
Between-Subjects phone-unlocks × Within-Subjects phone-unlocks	0.002	0.004	0.546	0.004	0.003	0.212
**R^2**	0.037			0.043		

The Relationship between Mood and Phone-Unlocks (Supplemental Table 2b)

Better mood was associated with higher phone-unlock counts across the study population during both the concurrent week (Between Subjects: β: -0.036; 95% CI [-0.055 to -0.016]; *p*<0.001), and subsequent week (Between Subjects: β: -0.026; 95% CI [-0.046 to -0.006]; *p*=0.012) . The opposite effect is noted when examining mood relative to one's baseline during the subsequent week (Within Subjects: β: 0.010; 95% CI [2e-04 to 0.020]; *p*=0.032).

Supplemental Table 2b. Model Results For Analyses Predicting Smartphone Unlock Counts by Mood

Using Weekly Mood to Predict:	Subsequent Week Phone-Unlocks			Concurrent Week Phone-Unlocks		
**Model Predictor**	**β**	**SE**	**p-Val**	**β**	**SE**	**p-Val**
Age	-0.031	0.001	**<0.001**	-0.031	0.001	**<0.001**
Gender [Female vs Male(ref)]	-0.235	0.021	**<0.001**	-0.234	0.021	**<0.001**
Gender [Genderqueer/Transgender vs Male(ref)]	-0.358	0.054	**<0.001**	-0.369	0.053	**<0.001**
Between-Subjects daily check-in	-0.026	0.010	**0.012**	-0.036	0.010	**<0.001**
Within-Subjects daily check-in	0.010	0.005	**0.032**	0.002	0.004	0.604
Between-Subjects daily check-in × Age	2e-04	3e-04	0.592	3e-04	3e-04	0.308
Between-Subjects daily check-in × Gender [Female vs Male(ref)]	0.009	0.009	0.325	0.014	0.009	0.098
Between-Subjects daily check-in × Gender [Genderqueer/Transgender vs Male(ref]]	-0.026	0.023	0.259	0.003	0.020	0.896
Between-Subjects daily check-in × Within-Subjects daily check-in	0.004	0.002	0.093	0.002	0.002	0.453
**R^2**	0.152			0.154		

Supplemental Results B: Analyses of day-to-day associations

The Relationship between Smartphone Use and Mood (Supplemental Table 3)

Association with Social-App Usage (Subsequent Day)

No associations between social app usage and subsequent daily mood were observed across the study population or when examining app usage relative to one's own baseline. Otherwise, results are largely similar to weekly models examining the same associations.

Association with non-Social-App Usage (Subsequent Day)

Results are largely similar to weekly models examining the same association. Higher non-social-app usage was associated with lower mood across the study population (Between Subjects: β: 0.071; 95% CI [0.053 to 0.089]; $p<0.001$), with the opposite effect observed when examining associations relative to one's own baseline (Within-Subjects; β: -0.034; 95% CI [-0.044 to -0.024]; $p<0.001$).

Supplemental Table 3. Model Results For Analyses Predicting Mood by Smartphone Use - Daily Lag

Predicting:	Subsequent Daily Mood from:					
	Social App Usage			Non-Social App Usage		
**Model Predictor**	**β**	**SE**	**p-Val**	**β**	**SE**	**p-Val**
Age	-0.010	0.001	**<0.001**	-0.010	5e-04	**<0.001**
Gender [Female vs Male(ref)]	0.077	0.014	**<0.001**	0.077	0.013	**<0.001**
Gender [Genderqueer/Transgender vs Male(ref)]	0.261	0.035	**<0.001**	0.252	0.034	**<0.001**
Between-Subjects social-app usage	0.016	0.010	0.118			
Within-Subjects social-app usage	0.008	0.005	0.092			
Between-Subjects social-app usage × Age	-3e-04	3e-04	0.341			
Between-Subjects social-app usage × Gender [Female vs Male(ref)]	-0.010	0.009	0.242			
Between-Subjects social-app usage × Gender [Genderqueer/Transgender vs Male(ref)]	-0.010	0.017	0.562			
Between-Subjects social-app usage × Within-Subjects social-app usage	0.001	0.001	0.521			
Between-Subjects non-social app usage				0.071	0.009	**<0.001**
Within-Subjects non-social app usage				-0.034	0.005	**<0.001**
Between-Subjects non-social app usage × Age				-4e-04	3e-04	0.140
Between-Subjects non-social app usage × Gender [Female vs Male(ref)]				0.011	0.008	0.145
Between-Subjects non-social app usage × Gender [Genderqueer/Transgender vs Male (ref)]				-0.010	0.019	0.579
Between-Subjects non-social app usage × Within-Subjects non-social app usage				0.001	0.002	0.546
**R^2**	0.020			0.024		

Association Between Mood and Device Usage at the Daily Level (Supplemental Table 4)

Association with Social-app Usage (Subsequent Day)

No significant predictive associations are seen for Between-Subject or Within-Subject daily check-in scores and social-app usage during the subsequent day.

Association with non-Social-app Usage (Subsequent Day)

Results are largely similar to models examining these associations at the weekly level. Better daily mood was associated with lower daily non-social-app usage across the study population (Between Subjects: β: 0.044; 95% CI [0.026 to 0.062]; *p*<0.001). The opposite was noted for daily mood relative to a participant's own baseline on non-social app usage during the subsequent day (Within-Subjects; β: -0.035; 95% CI [-0.049 to -0.021]; *p*<0.001).

Supplemental Table 4. Model Results For Analyses Predicting Smartphone Use by Mood - Daily Lag

Using Daily Mood to Predict:	Subsequent Day Social-App Usage			Subsequent Day non-Social App usage		
**Model Predictor**	**β**	**SE**	**p-Val**	**β**	**SE**	**p-Val**
Age	-0.012	0.001	**<0.001**	0.001	0.001	0.058
Gender [Female vs Male(ref)]	0.278	0.019	**<0.001**	0.063	0.019	**0.001**
Gender [Genderqueer/Transgender vs Male(ref)]	0.276	0.048	**<0.001**	0.197	0.049	**<0.001**
Between-Subjects daily questionnaire	0.002	0.008	0.802	0.044	0.009	**<0.001**
Within-Subjects daily questionnaire	0.003	0.007	0.662	-0.035	0.007	**<0.001**
Between-Subjects daily questionnaire × Age	2e-05	1e-04	0.905	-1e-04	2e-04	0.392
Between-Subjects daily questionnaire × Gender [Female vs Male(ref)]	-0.001	0.004	0.833	-0.001	0.004	0.773
Between-Subjects daily questionnaire × Gender [Genderqueer/Transgender vs Male(ref]]	0.003	0.011	0.793	-0.001	0.010	0.917
Between-Subjects daily questionnaire × Within-Subjects daily questionnaire	-0.002	0.001	0.139	-0.001	0.001	0.273
**R^2**	0.039			0.003		

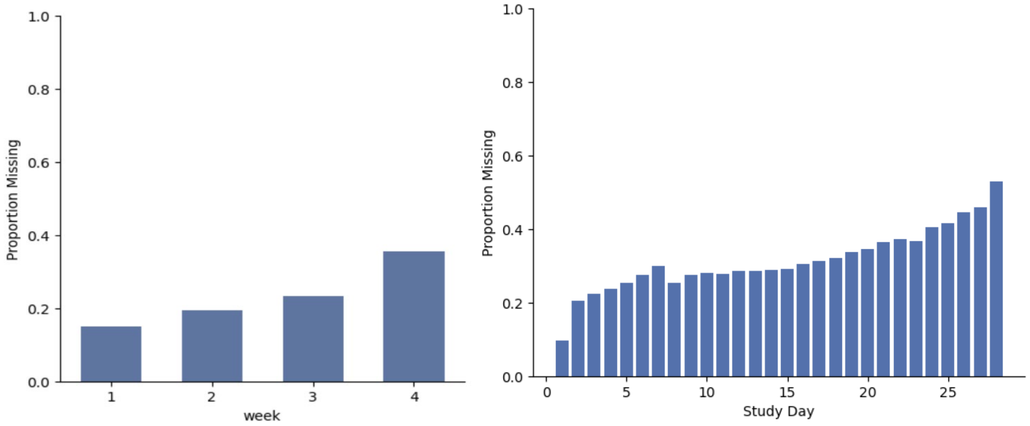

Supplemental Figure 1. Left: Proportion of participants for which mean daily check-in scores were missing due to inadequate coverage for the week in question. Right: Proportion of missing responses to the daily check-in questionnaires for each day of the 28-day study period.

Supplemental Methods A: Social Media Applications

Mobile Application Categorization

The following applications were categorized as “Social Media Apps” for the purpose of this study, based on their primary classification on the Android Play Store (https://play.google.com/store/apps/category/SOCIAL). All other applications were included in the non-social application category.

Bigo Live
Bigo Live Lite
Facebook
Facebook Lite
Hago
Helo
imo Lite
Instagram
Instagram Lite
Josh
Likee
Likee Lite
Litmatch
Moj
Moj Lite
OK: Social Network
PublicVibe
Reddit
Rizzle
Sharechat
Spark Live
StorySaver
StreamKar
Tango-Live Stream
Telegram
Tiki
TikTok
Twitter/X
Video Downloader for Instagram
VidStatus
VK: music, video, messenger

Supplemental Methods B: Survey Items

Survey Questions

Daily Mood Survey

ID	Text Type	Copy
DWB daily Q1-1	Question	In general, how have you been feeling over the last day?
		Very good
		Good
		Neutral
		Bad
		Very bad

Demographic Survey

ID	Text Type	Copy
DWB demo Q1	Question	What is your gender?
	Subtitle "Why we are asking this question"	This info will help researchers ensure good representation of all types of people in this study.
DWB demo A1-1	Answer	Female
DWB demo A1-2	Answer	Genderqueer/Gender Non-Conforming
DWB demo A1-3	Answer	Male

DWB demo A1-4	Answer	Trans Male/Trans Man
DWB demo A1-5	Answer	Trans Female/Trans Woman
DWB demo A1-6	Answer	Different Identity
DWB demo Q2	Question	What is your age?
	Subtitle "Why we are asking this question"	This info will help researchers ensure good representation of all types of people in this study.
DWB demo Q3	Question	What is your living situation?
	Subtitle "Why we are asking this question"	This info will help researchers ensure good representation of all types of people in this study.
DWB demo A3-1	Answer	Alone
DWB demo A3-2	Answer	With others
DWB demo Q4	Question	What is your current marital status?
	Subtitle "Why we are asking this question"	This info will help researchers ensure good representation of all types of people in this study.
DWB demo A4-1	Answer	Divorced
DWB demo A4-2	Answer	Married or Partnered
DWB demo A4-3	Answer	Never married
DWB demo A4-4	Answer	Separated
DWB demo A4-5	Answer	Widowed
DWB demo Q5	Question	How would you describe yourself?
	Subtitle "Why we are asking this question"	This info will help researchers ensure good representation of all types of people in this study.
DWB demo A5-1	Answer	Bisexual
DWB demo A5-2	Answer	Gay

DWB demo A5-3	Answer	Heterosexual or straight
DWB demo A5-4	Answer	Lesbian
DWB demo A5-5	Answer	Not listed above
DWB demo Q6	Question	Which of the following best describes you?
	Subtitle "Why we are asking this question"	This info will help researchers ensure good representation of all types of people in this study.
DWB demo A6-1	Answer	Hispanic or Latino/a
DWB demo A6-2	Answer	Not Hispanic or Latino/a
DWB demo Q7	Question	How would you describe yourself?
		Select all that apply
DWB demo A7-1	Answer	American Indian/Native American
DWB demo A7-2	Answer	Asian
DWB demo A7-3	Answer	Black/African American
DWB demo A7-4	Answer	Caucasian
DWB demo A7-5	Answer	Native Hawaiian/Pacific Islander
DWB demo A7-6	Answer	Unknown
DWB demo A7-7	Answer	Choose not to answer
DWB demo Q8	Question	Is English your primary language?
	Subtitle "Why we are asking this question"	This info will help researchers ensure good representation of all types of people in this study.
DWB demo A8-1	Answer	Yes
DWB demo A8-2	Answer	No
DWB demo Q9	Question	What is your primary language?
	Subtitle	Type below

DWB demo Q10	Question	How long have you been speaking English?
	Subtitle	Type below
DWB demo Q11	Question	What is your highest level of education?
	Subtitle "Why we are asking this question"	This info will help researchers ensure good representation of all types of people in this study.
DWB demo A11-1	Answer	High School
DWB demo A11-2	Answer	Associate’s Degree
DWB demo A11-3	Answer	Bachelor’s Degree
DWB demo A11-4	Answer	Graduate School
DWB demo A11-5	Answer	None of the above
DWB demo Q12	Question	Are you currently working?
DWB demo A12-1	Answer	Yes
DWB demo A12-2	Answer	No
DWB demo Q13	Question	What is your current occupational title?
	Subtitle	If retired, please indicate your former occupational title.
DWB demo Q14-1	Question	Do you identify as having a disability as defined under the [Americans with Disabilities Act] (https://adata.org/faq/what-definition-disability-under-ada)?
DWB demo Q14-2	Subtitle	The ADA defines a person with a disability as a person who has a physical or mental impairment that substantially limits one or more major life activities.
DWB demo A14-1	Answer	Yes
DWB demo A14-2	Answer	No
DWB demo A14-3	Answer	Prefer not to answer

DWB demo Q15	Question	Does your disability affect how you work?
	Subtitle "Why we are asking this question"	This info will help researchers ensure good representation of all types of people in this study.
DWB demo A15-1		Yes
DWB demo A15-2		No
DWB demo A15-3		Prefer not to answer
DWB demo Q16	Question	Thinking about your money situation, would you say you… (please choose one)
	Subtitle "Why we are asking this question"	This info will help researchers ensure good representation of all types of people in this study.
DWB demo A16-1		Can't make ends meet
DWB demo A16-2		Have just enough to get along
DWB demo A16-3		Are comfortable
DWB demo Q17	Question	Are you currently on Medicaid?
	Subtitle "Why we are asking this question"	This info will help researchers ensure good representation of all types of people in this study.
DWB demo A17-1		Yes
DWB demo A17-2		No
DWB demo Q18	Question	What is your Zip code?
	Subtitle "Why we are asking this question"	This info will help researchers ensure good representation of all types of people in this study.
DWB demo Q19	Question	Do you own a Fitbit?
	Subtitle	Select 'Yes' if you are able to wear it during the study.
		Yes

		No

Baseline Surveys

ID	Text Type	Copy
DWB baseline phq8 Q1-1	Pre-title	Over the last 2 weeks, how often have you been bothered by any of the following problems?
DWB baseline phq8 Q1-2	Question	Little interest or pleasure in doing things
		Not at all
		Several days
		More than half the days
		Nearly every day
DWB baseline phq8 Q2-1	Pre-title	Over the last 2 weeks, how often have you been bothered by any of the following problems?
DWB baseline phq8 Q2-2	Question	Feeling down, depressed, or hopeless
		Not at all
		Several days
		More than half the days
		Nearly every day
DWB baseline phq8 Q3-1	Pre-title	Over the last 2 weeks, how often have you been bothered by any of the following problems?
DWB baseline phq8 Q3-2	Question	Trouble falling or staying asleep, or sleeping too much
		Not at all

		Several days
		More than half the days
		Nearly every day
DWB baseline phq8 Q4-1	Pre-title	Over the last 2 weeks, how often have you been bothered by any of the following problems?
DWB baseline phq8 Q4-2	Questi	Feeling tired or having little energy
		Not at all
		Several days
		More than half the days
		Nearly every day
DWB baseline phq8 Q5-1	Pre-title	Over the last 2 weeks, how often have you been bothered by any of the following problems?
DWB baseline phq8 Q5-2	Question	Poor appetite or overeating
		Not at all
		Several days
		More than half the days
		Nearly every day
DWB baseline phq8 Q6-1	Pre-title	Over the last 2 weeks, how often have you been bothered by any of the following problems?
DWB baseline phq8 Q6-2	Question	Feeling bad about yourself—or that you are a failure or have let yourself or your family down
		Not at all
		Several days
		More than half the days

		Nearly every day
DWB baseline phq8 Q7-1	Pre-title	Over the last 2 weeks, how often have you been bothered by any of the following problems?
DWB baseline phq8 Q7-2	Question	Trouble concentrating on things, such as reading the newspaper or watching television
		Not at all
		Several days
		More than half the days
		Nearly every day
DWB baseline phq8 Q8-1	Pre-title	Over the last 2 weeks, how often have you been bothered by any of the following problems?
DWB baseline phq8 Q8-2	Question	Moving or speaking so slowly that other people could have noticed? Or the opposite—being so fidgety or restless that you have been moving around a lot more than usual
		Not at all
		Several days
		More than half the days
		Nearly every day
DWB baseline gad7 Q1-1	Pre-title	Over the last 2 weeks, how often have you been bothered by the following problem?
DWB baseline gad7 Q1-2	Question	Feeling nervous, anxious or on edge
		Not at all
		Several days
		More than half the days
		Nearly every day

DWB baseline gad7 Q2-1	Pre-title	Over the last 2 weeks, how often have you been bothered by the following problem?
DWB baseline gad7 Q2-2	Question	Not being able to stop or control worrying
		Not at all
		Several days
		More than half the days
		Nearly every day
DWB baseline gad7 Q3-1	Pre-title	Over the last 2 weeks, how often have you been bothered by the following problem?
DWB baseline gad7 Q3-2	Question	Worrying too much about different things
		Not at all
		Several days
		More than half the days
		Nearly every day
DWB baseline gad7 Q4-1	Pre-title	Over the last 2 weeks, how often have you been bothered by the following problem?
DWB baseline gad7 Q4-2	Question	Trouble relaxing
		Not at all
		Several days
		More than half the days
		Nearly every day
DWB baseline gad7 Q5-1	Pre-title	Over the last 2 weeks, how often have you been bothered by the following problem?
DWB baseline gad7 Q5-2	Question	Being so restless that it is hard to sit still
		Not at all
		Several days

		More than half the days
		Nearly every day
DWB baseline gad7 Q6-1	Pre-title	Over the last 2 weeks, how often have you been bothered by the following problem?
DWB baseline gad7 Q6-2	Question	Becoming easily annoyed or irritable
		Not at all
		Several days
		More than half the days
		Nearly every day
DWB baseline gad7 Q7-1	Pre-title	Over the last 2 weeks, how often have you been bothered by the following problem?
DWB baseline gad7 Q7-2	Question	Feeling afraid as if something awful might happen
		Not at all
		Several days
		More than half the days
		Nearly every day
DWB baseline promis sleep disturbance Q1	Pre-title	In the past 7 days...
	Question	My sleep was restless
		Not at all
		A little bit
		Somewhat
		Quite a bit
		Very much

DWB baseline promis sleep disturbance Q2	Pre-title	In the past 7 days...
	Question	I was satisfied with my sleep
		Not at all
		A little bit
		Somewhat
		Quite a bit
		Very much
DWB baseline promis sleep disturbance Q3	Pre-title	In the past 7 days...
	Question	My sleep was refreshing
		Not at all
		A little bit
		Somewhat
		Quite a bit
		Very much
DWB baseline promis sleep disturbance Q4	Pre-title	In the past 7 days...
	Question	I had difficulty falling asleep
		Not at all
		A little bit
		Somewhat
		Quite a bit
		Very much
DWB baseline promis sleep disturbance Q5	Pre-title	In the past 7 days...

	Question	I had trouble staying asleep
		Never
		Rarely
		Sometimes
		Often
		Always
DWB baseline promis sleep disturbance Q6	Pre-title	In the past 7 days...
	Question	I had trouble sleeping
		Never
		Rarely
		Sometimes
		Often
		Always
DWB baseline promis sleep disturbance Q7	Pre-title	In the past 7 days...
	Question	I got enough sleep
		Never
		Rarely
		Sometimes
		Often
		Always
DWB baseline promis sleep disturbance Q8	Pre-title	In the past 7 days...
	Question	My sleep quality was

		Very poor
		Poor
		Fair
		Good
		Very good
DWB baseline promis sleep impairment Q1	Pre-title	In the past 7 days...
	Question	I had a hard time getting things done because I was sleepy
		Not at all
		A little bit
		Somewhat
		Quite a bit
		Very much
DWB baseline promis sleep impairment Q2	Pre-title	In the past 7 days...
	Question	I felt alert when I woke up
		Not at all
		A little bit
		Somewhat
		Quite a bit
		Very much
DWB baseline promis sleep impairment Q3	Pre-title	In the past 7 days...
	Question	I felt tired
		Not at all

		A little bit
		Somewhat
		Quite a bit
		Very much
DWB baseline promis sleep impairment Q4	Pre-title	In the past 7 days...
	Question	I had problems during the day because of poor sleep
		Not at all
		A little bit
		Somewhat
		Quite a bit
		Very much
DWB baseline promis sleep impairment Q5	Pre-title	In the past 7 days...
	Question	I had a hard time concentrating because of poor sleep
		Not at all
		A little bit
		Somewhat
		Quite a bit
		Very much
DWB baseline promis sleep impairment Q6	Pre-title	In the past 7 days...
	Question	I felt irritable because of poor sleep
		Not at all

		A little bit
		Somewhat
		Quite a bit
		Very much
DWB baseline promis sleep impairment Q7	Pre-title	In the past 7 days...
	Question	I was sleepy during the daytime
		Not at all
		A little bit
		Somewhat
		Quite a bit
		Very much
DWB baseline promis sleep impairment Q8	Pre-title	In the past 7 days...
	Question	I had trouble staying awake during the day
		Not at all
		A little bit
		Somewhat
		Quite a bit
		Very much
DWB baseline promis emotional support Q1	Question	I have someone who will listen to me when I need to talk
	Subtitle	Select one
		Never
		Rarely

		Sometimes
		Usually
		Always
DWB baseline promis emotional support Q2	Question	I have someone to confide in or talk to about myself or my problems
	Subtitle	Select one
		Never
		Rarely
		Sometimes
		Usually
		Always
DWB baseline promis emotional support Q3	Question	I have someone who makes me feel appreciated
	Subtitle	Select one
		Never
		Rarely
		Sometimes
		Usually
		Always
DWB baseline promis emotional support Q4	Question	I have someone to talk with when I have a bad day
	Subtitle	Select one
		Never
		Rarely
		Sometimes

		Usually
		Always
DWB baseline SAS Q1	Pre-title	Indicate the degree to which you agree to the following using the scale below
	Question	I miss planned work due to smartphone use
		Strongly disagree
		Disagree
		Weakly disagree
		Weakly agree
		Agree
		Strongly agree
DWB baseline SAS Q2	Pre-title	Indicate the degree to which you agree to the following using the scale below
	Question	I have a hard time concentrating in class, while doing assignments, or while working due to smartphone use
		Strongly disagree
		Disagree
		Weakly disagree
		Weakly agree
		Agree
		Strongly agree
DWB baseline SAS Q3	Pre-title	Indicate the degree to which you agree to the following using the scale below
	Question	I feel pain in the wrists or at the back of the neck while using a smartphone
		Strongly disagree

		Disagree
		Weakly disagree
		Weakly agree
		Agree
		Strongly agree
DWB baseline SAS Q4	Pre-title	Indicate the degree to which you agree to the following using the scale below
	Question	I won’t be able to stand not having a smartphone
		Strongly disagree
		Disagree
		Weakly disagree
		Weakly agree
		Agree
		Strongly agree
DWB baseline SAS Q5	Pre-title	Indicate the degree to which you agree to the following using the scale below
	Question	I feel impatient and fretful when I am not holding my smartphone
		Strongly disagree
		Disagree
		Weakly disagree
		Weakly agree
		Agree
		Strongly agree

DWB baseline SAS Q6	Pre-title	Indicate the degree to which you agree to the following using the scale below
	Question	I have my smartphone in my mind even when I am not using it
		Strongly disagree
		Disagree
		Weakly disagree
		Weakly agree
		Agree
		Strongly agree
DWB baseline SAS Q7	Pre-title	Indicate the degree to which you agree to the following using the scale below
	Question	I will never give up using my smartphone even when my daily life is already greatly affected by it.
		Strongly disagree
		Disagree
		Weakly disagree
		Weakly agree
		Agree
		Strongly agree
DWB baseline SAS Q8	Pre-title	Indicate the degree to which you agree to the following using the scale below
	Question	I am constantly checking my smartphone so as not to miss conversations between other people on Twitter or Facebook
		Strongly disagree
		Disagree

		Weakly disagree
		Weakly agree
		Agree
		Strongly agree
DWB baseline SAS Q9	Pre-title	Indicate the degree to which you agree to the following using the scale below
	Question	I use my smartphone longer than I had intended
		Strongly disagree
		Disagree
		Weakly disagree
		Weakly agree
		Agree
		Strongly agree
DWB baseline SAS Q10	Pre-title	Indicate the degree to which you agree to the following using the scale below
	Question	The people around me tell me that I use my smartphone too much.
		Strongly disagree
		Disagree
		Weakly disagree
		Weakly agree
		Agree
		Strongly agree
DWB baseline PSS Q1	Question	In the last month, how often have you been upset because of something that happened unexpectedly?

		Never
		Almost Never
		Sometimes
		Fairly Often
		Very Often
DWB baseline PSS Q2	Question	In the last month, how often have you felt that you were unable to control the important things in your life?
		Never
		Almost Never
		Sometimes
		Fairly Often
		Very Often
DWB baseline PSS Q3	Question	In the last month, how often have you felt nervous and “stressed?”
		Never
		Almost Never
		Sometimes
		Fairly Often
		Very Often
DWB baseline PSS Q4	Question	In the last month, how often have you felt confident about your ability to handle your personal problems?
		Never
		Almost Never
		Sometimes

		Fairly Often
		Very Often
DWB baseline PSS Q5	Question	In the last month, how often have you felt that things were going your way?
		Never
		Almost Never
		Sometimes
		Fairly Often
		Very Often
DWB baseline PSS Q6	Question	In the last month, how often have you found that you could not cope with all the things that you had to do?
		Never
		Almost Never
		Sometimes
		Fairly Often
		Very Often
DWB baseline PSS Q7	Question	In the last month, how often have you been able to control irritations in your life?
		Never
		Almost Never
		Sometimes
		Fairly Often
		Very Often
DWB baseline PSS Q8	Question	In the last month, how often have you felt that you were on top of things?

		Never
		Almost Never
		Sometimes
		Fairly Often
		Very Often
DWB baseline PSS Q9	Question	In the last month, how often have you been angered because of things that were outside of your control?
		Never
		Almost Never
		Sometimes
		Fairly Often
		Very Often
DWB baseline PSS Q10	Question	In the last month, how often have you felt difficulties were piling up so high that you could not overcome them?
		Never
		Almost Never
		Sometimes
		Fairly Often
		Very Often
DWB baseline BFI10 Q1	Question	I see myself as someone who is reserved
		Disagree strongly
		Disagree a little
		Neither agree nor disagree
		Agree a little

		Agree strongly
DWB baseline BFI10 Q2	Question	I see myself as someone who is generally trusting
		Disagree strongly
		Disagree a little
		Neither agree nor disagree
		Agree a little
		Agree strongly
DWB baseline BFI10 Q3	Question	I see myself as someone who tends to be lazy
		Disagree strongly
		Disagree a little
		Neither agree nor disagree
		Agree a little
		Agree strongly
DWB baseline BFI10 Q4	Question	I see myself as someone who is relaxed, handles stress well
		Disagree strongly
		Disagree a little
		Neither agree nor disagree
		Agree a little
		Agree strongly
DWB baseline BFI10 Q5	Question	I see myself as someone who has few artistic interests
		Disagree strongly
		Disagree a little

		Neither agree nor disagree
		Agree a little
		Agree strongly
DWB baseline BFI10 Q6	Question	I see myself as someone who is outgoing, sociable
		Disagree strongly
		Disagree a little
		Neither agree nor disagree
		Agree a little
		Agree strongly
DWB baseline BFI10 Q7	Question	I see myself as someone who tends to find fault with others
		Disagree strongly
		Disagree a little
		Neither agree nor disagree
		Agree a little
		Agree strongly
DWB baseline BFI10 Q8	Question	I see myself as someone who does a thorough job
		Disagree strongly
		Disagree a little
		Neither agree nor disagree
		Agree a little
		Agree strongly
DWB baseline BFI10 Q9	Question	I see myself as someone who gets nervous easily

		Disagree strongly
		Disagree a little
		Neither agree nor disagree
		Agree a little
		Agree strongly
DWB baseline BFI10 Q10	Question	I see myself as someone who has an active imagination
		Disagree strongly
		Disagree a little
		Neither agree nor disagree
		Agree a little
		Agree strongly
